# “My right-hand man” versus “We barely make use of them”: change leaders talking about educational scientists in curriculum change processes—a Membership Categorization Analysis

**DOI:** 10.1007/s10459-019-09894-5

**Published:** 2019-05-08

**Authors:** Floor Velthuis, Esther Helmich, Hanke Dekker, Tom Koole, A. Debbie C. Jaarsma

**Affiliations:** 1 Center for Education Development and Research in Health Professions (CEDAR), LEARN, University Medical Center Groningen, University of Groningen, Antonius Deusinglaan 1, 9713 AV Groningen, The Netherlands; 2 Communication and Information Studies, University of Groningen, Groningen, The Netherlands; 3 School of Human and Community Development, University of the Witwatersrand, Johannesburg, South Africa

**Keywords:** Educational scientists, Health professions education scholarship units, Curriculum change, Linguistic analysis, Membership Categorization Analysis

## Abstract

Health professions education scholarship units (HPESUs) are increasingly becoming a standard for medical schools worldwide without having much information about their value and role in actual educational practices, particularly of those who work in these units, the educational scientists. We conducted a linguistic analysis, called Membership Categorization Analysis, of interviews with leaders of recent curriculum changes to explore how they talk about educational scientists in relation to these processes. The analysis was conducted on previously collected interview data with nine change leaders of major undergraduate medical curriculum change processes in the Netherlands. We analyzed how change leaders categorize HPESUs and educational scientists (use of category terms) and what they say about them (predicates). We noticed two ways of categorizing educational scientists, with observable different predicates. Educational scientists categorized by their first name were suggested to be closer to the change process, more involved in decisional practices and positively described, whereas those described in more generic terms were represented in terms of relatively passive and unspecified activities, were less explicit referenced for their knowledge and expertise and were predominantly factually or negatively described. This study shows an ambiguous portrayal of educational scientists by leaders of major curriculum change processes. Medical schools are challenged to establish medical curricula in consultation with a large, diverse and interdisciplinary stakeholder group. We suggest that it is important to invest in interpersonal relationships to strengthen the internal collaborations and make sure people are aware of each other’s existence and roles in the process of curriculum development.

## Introduction

Health professions education scholarship units (HPESUs) (Varpio et al. 2017a), are increasingly becoming a standard for medical schools worldwide; however, information about roles and contributions of their organizational members in actual educational practices is limited. The available literature focuses predominantly on (successes of) research and scholarship contributions (Arnold 2004; Elam 2004; Gruppen 2004; Hodges 2004; Irby et al. 2004; Norman 2011; van der Vleuten et al. 2004; Varpio et al. 2014; Varpio et al. 2017a; Wolf et al. 2004), whereas, in contrast, the more practical, ‘service’ related roles within the local institutional context (supporting the advancement of medical programs), remain largely understudied. The continuous growth of new HPESUs is argued to be evidence of their value in itself (Gruppen 2008), and it is assumed that their members have a role in supporting curricular change processes (Davis et al. 2005). However little is known about what their value and roles looks like in actual practices. To start exploring these roles, our study focuses on one particular organizational member of these HPESUs, the educational scientists.

Similar to Varpio et al. (2017a), we use the name health professions education scholarship units, noting that various other designations could be found in the literature, such as department, office, division, center, extra-departmental units, and other name variations alike (Davis et al. 2005; Gruppen 2008; Varpio et al. 2014; Varpio et al. 2017a). What they have in common is that members of these units try to find a balance between delivering a service to their local, institutional context, and contributing to a broader, international research agenda (Albert et al. 2007; Gruppen 2008; van der Vleuten 2014). In the scarce body of medical educational literature that touches upon the more’service’ related roles, HPESUs and its members are characterized as valuable resources (Davis et al. 2005; Varpio et al. 2017b). They are considered to be *‘an essential requirement for any medical school’,* having a role as *“service providers, helping (…) with aspects of teaching and learning, advising, providing expertise and offering support.”* (Davis et al. 2005, p. 674 and 669). Within these units Varpio et al. (2017a) identified three common roles; the clinician educators, research scientists, and administrative leaders. We focus on the role of research scientists, however, in this article we refer to this group as educational scientists, as we believe this term resonates better with our setting.

Educational scientists are trained in social and behavioral disciplines like education, pedagogy and (educational) psychology (Davis et al. 2005; Gruppen 2008; van der Vleuten 2014; Varpio et al. 2017a). They usually engage both in scholarship as well as service activities (Varpio et al. 2017a), however, the balance of time spend on service or scholarship varies per institute (Varpio et al. 2017b). In contrast to those ‘delivering’ the curriculum, i.e. teachers with (pre)clinical curriculum-content knowledge, educational scientists have specific knowledge about what a medical curriculum should look like in terms of didactics, teaching methods, assessment and other educational aspects. However, apart from the assumed value, we do not know much about the involvement of such educational scientists, specifically during curriculum change processes.

In an earlier study, we interviewed change leaders responsible for the enactment of a major undergraduate medical curriculum change process. We found that dealing with a large and diverse group of stakeholders involved was one of the main challenges (Velthuis et al. 2018). While working with these data we got interested by the language used by the change leaders when describing collaborations during the change process, specifically when talking about the group of educational scientists. Combined with anecdotal evidence, at least within the Netherlands, suggesting that educational scientists hold ambivalent positions in medical schools, we decided to explore this in more detail. Therefore, to get a better insight into the roles and conceptions of educational scientists within HPESUs during a curriculum change process, we conducted a linguistic analysis on the previously collected interview data (Velthuis et al. 2018).

In this study we wanted to better understand how educational scientists are perceived by change leaders, addressing the question how change leaders represent and talk about educational scientists in an interview about a major curriculum change process. To reach our goal, we used a novel method in medical educational literature, called Membership Categorization Analysis. A closer, detailed, look at language used by those leading the change process provides insight into how they represent educational scientists in the context of curriculum changes, and how leaders—by doing so—construct a specific social reality. The value of such an analysis is that it reveals their assumptions about educational scientists as social actors in this particular process (Stokoe 2012).

The language people use is a way of representing the world the speaker lives in. Language provides people with methods—such as words, phrases and intonations—by which they construct their realities. For example, by presenting something as a fact (e.g. ‘it is a pleasant hospital’) we construct a different reality than by representing it as an opinion (e.g. ‘I find it a pleasant hospital’). Also, we can choose to present an event as ordinary or as exceptional, and in a similar manner, a person can be referred to in different ways (e.g. ‘this man’, ‘my brother’, ‘the doctor’, ‘he’) each of which establishes a different version of reality. Linguistic choices are methods by which we bring about different realities (Ten Have 2004; Potter 1996; Edwards 1997). In this study we use language, and in particular the linguistic means for categorizing persons, as a lens to deepen our understanding of how people conceptualize others in relation to themselves and the processes in which they are involved.

## Method

The Dutch Association for Medical Education (NVMO) ethical review board approved this study (number: 592).

### Participants

Nine change leaders who lead or led the most recent major undergraduate medical curriculum change processes in one of the eight University Medical Centers (UMCs) in the Netherlands were included in this study. In one institute two leaders were identified, leading to nine participants (1 female, 8 male). In our research, we define ‘major curriculum changes’ as: *“changes that [are] not about the yearly, regular adjustments at course level, but were centrally organized, intentionally initiated change projects that impacted the entire curriculum and organization involved in the curriculum.*”(Velthuis et al. 2018, p. 1504). Assigned, and delegated by the Dean/Board of Directors, the change leaders were responsible for the change process, which distinguishes them from ‘important others members’ in the change process. They all (had) fulfilled leading positions in (pre)clinical or research departments, and had been involved in medical education and the medical school in various positions, for many years.

### Design

Our previous publication based on this data showed, among others, that change leaders faced the challenge of dealing with a large and diverse group of stakeholders (Velthuis et al. 2018), of which educational scientists were one. As we were interested in the language used when referring to educational scientists as a resource in curriculum changes, we conducted a Membership Categorization Analysis on this previously collected data. The semi-structured, individual face-to-face interviews were conducted by FV between December 2015 and April 2016, lasting 1.5 to 2 h. Participants were invited to talk about different themes and topics related to curriculum change, such as who were important stakeholders for them in the process to enact change, and how they dealt with them, the accelerators and decelerators of the process, (personal) challenges and lessons learned. Interviews were audio-recorded, and subsequently transcribed and anonymized.

### Context of educational scientists

In the Netherlands, educational scientists are employed in a HPESU of which exact names and organizational structures vary depending on the organizational context to which they are aligned (e.g. some are institutes or departments within a medical school, others belong to hospital division structures, etc.). However, overall, the educational scientists have similar roles in doing research and providing services to the local community related to, but not exclusively, didactics, curriculum design, teaching methods and assessment. With a few exceptions, these individuals neither teach in the medical program, nor provide patient care. They are primarily entrusted with the task to support the medical program(s) and its teaching community as advisors, and/or do research.

### Data analysis

#### Stance and orientation

Linguistic analyses, an umbrella term for studies that analyze language use, focus on “*talk and texts as social practices, and on the resources that are drawn on to enable those practices.*”(Potter 1996a, p. 7). Linguistic analyses are constructivist in that they assume ‘*that basic assumptions with regard to being, self and the world are constructed by individuals living in a historical and cultural context which is produced and reproduced by their speech acts.”* (Pedersen 2011, p. 673). This also means that, in line with Ahearn (2001), we perceive language to be a form of social action, in which “*meanings are co*-*constructed by participants, emergent from particular social interactions.”* (p. 111). Therefore, with language we create and constitute our realities.

In line with Ten Have (2004), we take the ethnomethodological perspective that sees social reality as produced by methods and practices of participants in that reality. This perspective assumes a continuity between interviewee practices within and outside the interview. Thus interviews reflecting on a reality (the curriculum change process) as well as that referred reality itself, are part of one and the same reality. Therefore, we assume a continuum between the language an interviewee uses to construct representations of educational scientists during the interviews and ways they interact and collaborate with educational scientists. Both *what* and *how* the respondents say about the educational scientists informs us about the representations of, as well as the interactions with this group during the change process.

Suiting our goal to understand how change leaders represent educational scientists and talk about them in the interviews, we conducted a particular linguistic analysis, called *Membership Categorization Analysis* (Day 2012; Stokoe 2012; Ten Have 2004). Membership Categorization Analysis (MCA) is an ethnomethodological approach that particularly focuses on identifying ‘categories’ speakers use to make sense of others and themselves in social interactions in our daily lives (for examples see analytical procedure below) (Day 2012; Stokoe 2012; Ten Have 2004). When people describe others, they use categories, and by using categories, and so called category-bound predicates (statements about/related to the person one is referring to), we show that we recognize people *“as certain sorts of people”* (Day 2012, p. 1). It is suggested that *“categories store ‘a great deal of the knowledge that members of a society have about the society’* (Sacks 1989) *… Each category carries a different set of category*-*bound activities, predicates, or rights and obligations that are expectable for an incumbent of that category to perform or possess* (Stokoe 2012, p. 282). Thus, using categories is an important resource for people in their dealing with each other, and since language is one of the primary ways we ‘deal’ with each other, MCA focuses on language use in naturally occurring speech, such as conversations and speeches, but also written pieces of text (Day 2012).

#### Analytical procedure

Our first step was to create a so called collection of the interview data we wanted to analyze (Stokoe 2012). This collection consisted of all the category *terms* referring to HPESUs, educational scientists and their corresponding *predicates*. Category terms are words or word combinations used to refer to someone/a group. As an example, ‘educational specialist’, ‘employees of the medical educational department’, and ‘support staff’, are all category terms for potentially the same person/group. Additionally, a predicate contains the characteristics, actions and activities ascribed to the person or group (the category term), and therefore provides information about the subject it refers to (Day 2012). For example, in the following sentence ‘The supporters’ is the category term, followed by the predicate in *italics*: ‘The supporters *are all the people who are involved in education but do not teach themselves’.* Together, various category terms could form one category. We stored the collection of category terms and corresponding predicates in Excel. This program was also used for further analysis.

As MCA, like various other analyses of qualitative data, starts as an open, explorative process, two researchers, FV and HD, first made themselves familiar with the data by reading and re-reading, and made observations about the various category terms and predicates (Stokoe 2012). The observations were discussed with the entire team, having TK as linguistic analysis specialist shaping the process. Subsequently, more detailed observation rounds led to working hypotheses on what was said about educational scientists. The hypotheses where further shaped by team discussions, resulting in the final results presented below.

### Reflections

Our interdisciplinary research team consisted of researchers having various backgrounds, providing a range of different perspectives on the topic of study. FV, who conducted the interviews, has a background in social psychology, and is interested in the use of language to construct our worlds. EH is an elderly care physician, experienced qualitative researcher, and clinical educator. HD is a senior educational scientist chairing a task group on education innovation during a major curriculum change process. TK is a professor in language and social interaction, with expertise in MCA and other linguistic analyses, who guided the analysis. AJ is a professor in health professions education research and directs a HPESU, staffing, amongst others, educational scientists. These various positions led us to experience and look at the research subject differently. During our team discussions, we deliberately built on our diverging perspectives, e.g. personal experiences with either being part of a group of educational scientists, or as teachers/leaders working together with educational scientists in curriculum change processes.

In 8 out of 9 interviews, FV conducted the interviews with participants from medical schools different from her own school, and had never met them upfront. Only in her own school she formally knew the participant, however, as FV had not been actively involved in the change process in this school they had never talked before. For the team members AJ and HD this participant was known, and HD had worked with this person on a regular basis during the change process. The participant did not consider this to be a problem and explained to feel free to speak about the process from his own perspective. In two other cases AJ and the participants knew each other, however, also in these cases the participants did not consider this a limitation to speak freely to FV. As FV carefully anonymized the transcripts, the participants and their institutes were unrecognizable for the team members.

### Translations

The interviews were conducted in Dutch. Therefore, as a Dutch team, we could all be deeply involved in a rich, in-depth analysis of the data. Having skilled English writers in our team, we translated some quotes for this article after the analysis. In case of doubt we consulted a bilingual colleague.

## Results

The way HPESUs and educational scientists were mentioned and described varied across and within the interviews. We will start with the HPESUs, followed by the use of category terms to refer to educational scientists within these units.

### HPESUs

‘Educational institute’ was the most common category term used to refer to HPESUs, and in various interviews respondents also used the local name.

For some respondents the *“educational world”* (P7, P8) was another, and different world compared to what they were used to. Compared to working in a hospital, the educational institute was described as *‘a very different world, that is different from the way you are used to work with each other in the hospital’*. This referred for example to having a more *‘more gamma*-*ish’* way of looking at reality, and having *‘much longer mutual consultation[s]’*. (P7). This participant did a master in education to *‘better understand the scene’* and *‘learn the language of the people with whom you are going to work, that is a different language and a different way of thinking.’ (P7).* Another one warned that educational departments could become an *‘ivory tower’* (P6), a feeling that was reinforced by the location the educational institute *‘at the far end of the corner of the hospital*.’ (P3)

### The use of category terms for educational scientists

We observed two ways of categorizing educational scientists by respondents. One way, applied by three respondents, was to use *person names* that explicitly refer to individual persons called by their first name. The another way, and observed in all interviews, was using *more generic category terms* which contain information related to educational scientists’ academic backgrounds and expertise/knowledge (“*educational scientist(s)*”, “*assessment experts*”), tasks/functions (“*people from faculty development*”), supportive role (“*people who are supportive in education*”, “*the supporters*”) and their belonging to the educational departments (“*people from the educational institute”, “employees of the educational institute”*). Most of the generic category terms were plural, assuming a collection of people, apart from a small number of exceptions such as “an educational scientist”.

Hereafter, we will describe these two ways of categorizing in more detail; first providing a general impression of what has been said about the category term, followed by a description of our observed differences in proximity to the change process/change leader, and differences in evaluation.

To ensure anonymity, names in the examples are fictitious.

### Person name category and the corresponding predicates

When looking more closely to the predicates that respondents related to those called by name, we saw that much that has been said about these individuals was rather specific, and explicitly referring to their knowledge and expertise as an educational scientist, for example:

“That is also Richard’s expertise as educational scientist, he wants to be more definite about [the route and goals], much more [than I do].” (R3)

“Peter (…) he knows what it should be like with this [particular type of educational method]. … He is leading the way in developing this [particular type of educational method]”] (R6)

#### Proximity to the process and/or change leader

By calling individuals personally by name change leaders presented these individuals as known to them as persons and as working closely with the change leader. More specifically, these individuals were also presented as actively engaged in decisive moments in the change process. They were for example part of a group of people “*where actually the real decisions are made” (R3)*, or who created the first draft of the new curriculum:

“David … is a senior educational scientist who also played an important role in other curriculum change processes. … The [curriculum plan] was made with the help of David.” (R4)

This proximity was also shown in the expressions such as a specific person being someone’s “*right*-*hand man” (R3)*.

#### Evaluation

Individuals called by name were consistently positively represented. The respondents explicitly used positive terms when ascribing personal characteristics, such as in this example representing Peter to be well informed:

“A great educational expert. … I really want Peter to be involved because I know that he has very good ideas, because he knows what is going on in the world.” (R6)

The only little more critical remark was found in the same case, where educational scientist Peter was described to be part of the change leader’s important stakeholder group, sitting in a boat but not being allowed to row, as he was going to steer in the direction of ideas that were beautiful, ‘*but not feasible’* (R6).

### Generic category and the corresponding predicates

When looking more closely to the predicates that respondents used in relation to the generic category terms, we found that much that was said about educational scientists was about what they did or were supposed to do in the process. What educational scientists contributed was related to activities such as providing support, giving advice and thinking along. Participants did not provide much more details about what this exactly involved or meant. Generally, these activities seemed to be passive, such as executing and following orders and tasks, rather than referring to educational scientists being in more pro-active, leading or decisive roles, as were the individuals for which change leaders used personal category terms. Like in this example where the task was presented as something that was expected from educational scientists and was also given to them:

“(…) and the educational scientists have to help with creating the [student] assignments, and they get those from various groups.” (R3)

With few exceptions, what was said about these generically categorized groups of educational scientists remained relatively unspecified, such as in the following example where the teachers were emphasized to be the main group, having the educational scientist ‘in support of’:

“So the project groups are mainly borne by teachers. It is mainly teachers, and besides that - in support of - there is an educational scientist.” (R9)

Although some category terms contained words suggesting expertise and knowledge of education, not so much of what was said about this group related to this expertise or to how this knowledge was applied or used in the curriculum change process.

#### Proximity to the change process and/or change leader

In some cases, educational scientists referred to with generic terms were mentioned to be consulted if other people experienced difficulties regarding educational issues, but one-on-one *“we barely make use of them”* (P6), or were only involved, “*if considered necessary”* (R1). Rather than being presented as a structural part of the process, educational scientists were in these conversations represented as consultants-on-demand. This minimal involvement sets the impression of a distance to the process and to the change leader.

In some other cases, change leaders stated that educational scientists were part of internal educational committees, which suggests a more closely involved position to the change process:

“There is of course - I forgot to mention but that speaks for itself - an educational scientist in our committee, and this person has written the semester guidelines.” (R2)

In this example, the educational scientist has a relatively clear activity: writing semester guidelines. However, apart from this example, similarly to what was previously mentioned, on many occasions it remained unspecified what they exactly did in these committees.

Only in very few predicates, some more explicit proximity to the change leader and the process was suggested, such as this example illustrates with ‘around me’:

“And of course I had some driven educational scientists around me who said things like: ‘this is not okay and good enough, too little change.’” (R2).

#### Evaluation

Compared to the consistently positive predicates related to those called by name, the predicates associated with the generic category terms were either more factual/neutral, or were explicitly negative.

In the negative expressions, educational scientists were for example associated with causing delays because of poor communication between them and another group, or because they did not do what the change leader expected of them. In addition, sometimes direct frustrations about their performances and characteristics/attitudes were expressed:

“Well, I have not become very impressed by the general quality of the educational support and the pace in which this goes. And I could express that in even much much stronger terms.” (R8).

“People who strongly advocate for [this type of education]. (…) Especially when it is posited with presumed authority like: I am the educational scientist here, so I know how it is and we do it all wrong.” (R5)

## Discussion

This study explored how change leaders represent and talk about educational scientists in a curriculum change process, using MCA, a novel linguistic analysis method in health professions education research. Doing this analysis showed us how change leaders make sense of their reality (Day 2012), and describe their world (Stokoe 2012), by using different categories, terms and qualifications for a group that is by us, and in literature, ‘just’ categorized as educational scientists. The results revealed a diverse array of perspectives about this group, ranging from barely-not-being-included in the process to notions of strong one-on-one collaborations. Our findings suggest that educational scientists who were referred to in a more personal way by the change leader, are more closely involved in the change process compared to those referred to in more generic terms. Without claiming that those mentioned in more generic terms are of less value for the change leader, it is at least remarkable how explicitly positive change leaders talk about these personally known individuals compared to the more factual/neutral, and even negative expressions related to generically categorized educational scientists. Our results led us to believe that in various institutes these educational scientists do not always seem to be optimally visible, engaged and connected, which is inconvenient both for the units and educational scientists itself, as well as for the broader organization in general.

This paper contributes to the literature on curriculum change processes, specifically focusing on the involvement of a particular stakeholder group, the educational scientists. Although much is written about the importance of stakeholder involvement in curriculum change in general (Edwards et al. 2017; Bland et al. 2000), and about the roles of teachers (Venance et al. 2014) and students (Atkins et al. 1998) particularly, no attention have been paid to this group of educational scientists in HPESUs that are a common, and growing phenomena (Davis et al. 2005; Varpio et al. 2017a). Our results therefore enhance our understanding of the involvement of this particular group, and shows us that differences might occur in various change processes regarding their involvement and wider collaborations within medical schools.

Our findings resonate with findings of Hu et al. (2015). We see a similarity in the struggle of being a non-clinically trained individual in a predominantly clinically-trained environment: trying to be visible and being similarly credited as your clinically trained colleagues if you do not have a medical background in a medical school (Hu et al. 2015). All change leaders in our study had (pre)clinical backgrounds, and some talked explicitly negative about educational scientists. Elaborating on the work of Hu et al. (2015) our findings suggest that being known as a person is important to overcome this non-clinical barrier, to be able to show your added value and being credited as a serious partner. Being this non-clinical group ‘far away in the corridor of the hospital’ does not help in creating visibility and becoming such a partner, making it more difficult to be involved.

Our findings also correspond with another linguistic study that showed that explicitly identifying a particular person or a group in a speech act is never a prelude to criticism. In their study, Koole (2003), found that criticizing a particular group was done by using more anonymized references, (e.g. the oppressors), whereas when it was talked about a group that had done something positive, they were explicitly named or identified (e.g. the Canadians). This is an interesting discursive strategy indicating that criticizing combines easier with anonymized referents than identifiable referents, even in cases like ours, where participants were ensured that the data will be anonymized and treated confidentially.

As Stivers et al. (2007) describe, the use of particular references *‘is not just, indeed not primarily, about giving and receiving information but about navigating social relations.’* (p. 19). When the use of personal references reveals something about the relationship between the change leader and the educational scientist that is referred to, referring to someone by name establishes a closer relationship, compared to those referred to in general terms. This is apparent also in our finding that the personally identified educational scientists were more positively described and associated to be closer involved in the process. In line with the ethnomethodological perspective (Ten Have 2004), we assume that a continuity exists between the language an interviewee uses to construct representations of people during the interviews and ways they interact and collaborate with them. Therefore, the analysis of the language used in the interviews does not only gives us insight in the practices change leaders use in answering interview questions, but also in the practices they use in dealing with the staff of HPESUs.

Building on our interpretation of the data, we suggest that it is important to invest in interpersonal relationships to strengthen the internal collaborations and make sure people are aware of each other’s existence, and roles in the process of curriculum development. One could think of organizing activities, such as educational seminars in which educational scientists and faculty (such as curriculum leaders and teachers) come together and share research ideas and practical implications. This, and other imaginable activities that foster collaborations, might facilitate the visibility and enhance the comprehension of each other’s language and ways of thinking, and therefore, ultimately strengthen the relationships.

### Reflections/limitations

In the present study, we analyzed naturally occurring conversations (interview data) which suits the method of MCA that could be applied to all kinds of speech related data (Day 2012). During the interviews we asked all the respondents about the important stakeholders in the change process, and deliberately elaborated on their answers. Analyzing these data gave us the opportunity to explore what is being said about educational scientists spontaneously. We could have asked change leaders explicitly about their opinion on educational scientists, but that might have resulted in socially acceptable answers. Therefore we assume that the most significant information was included in the existing data containing information that had come up unrestrained and spontaneously.

Reflecting on our team composition, and inherent to qualitative research, other researchers might have focused their analysis on other aspects of the conversations, possibly resulting in different interpretations. By critically examining the various points of view regarding the data and interpretations, and at the same time keeping the interview context in mind in which utterances were placed, we believe that we were able to come to a valuable representations of the data. We encourage other researchers to try additional approaches in this area of interpersonal, professional relationships that is ripe for further sociological research.

One gender remark we would like to make is the fact that most UMCs choose to have a male change leader (8 out of 9), and that the educational scientists that where personally referred to, were only males. Therefore, the results might not be representative for other institutes. Having males predominantly in leadership positions resonates with the well-known trends of gender disparity in leadership positions (Hoyt 2010). However, why the participants only personally identify male educational scientists cannot be answered based on our data. Future studies might want to study whether gender indeed plays a role or not in collaborations between curriculum leaders and educational scientists.

Additionally, to get a more comprehensive view of the representations of other stakeholders (either by a leader of the process or from other stakeholders), and what this indicates for the interpersonal relationships and collaborations, future research might want to explore the perspectives of other stakeholders in the change process. Finally, although the results of our study refer to, and originate from, specific local contexts that are—in itself—perhaps not directly transferable to other contexts, we do believe that the overall message of ambiguity towards educational scientists should invite readers to reflect on their own particular setting.

## Conclusion

This study shows an ambiguous portrayal of educational scientists by leaders of major curriculum change processes. Medical schools are challenged to establish medical curricula in consultation with a large, diverse and interdisciplinary stakeholder group. We suggest that it is important to invest in relationships to strengthen the internal collaborations and make sure people are aware of each other’s existence and roles in the process of curriculum development.
